# Comprehensive Modeling and Discovery of Mebendazole as a Novel TRAF2- and NCK-interacting Kinase Inhibitor

**DOI:** 10.1038/srep33534

**Published:** 2016-09-21

**Authors:** Zhi Tan, Lu Chen, Shuxing Zhang

**Affiliations:** 1 Integrated Molecular Discovery Laboratory, Department of Experimental Therapeutics, The University of Texas MD Anderson Cancer Center, Houston, TX 77030, USA; 2 The University of Texas Graduate School of Biomedical Sciences, Houston, TX 77030, USA

## Abstract

TRAF2- and NCK-interacting kinase (TNIK) represents one of the crucial targets for Wnt-activated colorectal cancer. In this study, we curated two datasets and conducted a comprehensive modeling study to explore novel TNIK inhibitors with desirable biopharmaceutical properties. With Dataset I, we derived Comparative Molecular Similarity Indices Analysis (CoMSIA) and variable-selection k-nearest neighbor models, from which 3D-molecular fields and 2D-descriptors critical for the TNIK inhibitor activity were revealed. Based on Dataset II, predictive CoMSIA-SIMCA (Soft Independent Modelling by Class Analogy) models were obtained and employed to screen 1,448 FDA-approved small molecule drugs. Upon experimental evaluations, we discovered that mebendazole, an approved anthelmintic drug, could selectively inhibit TNIK kinase activity with a dissociation constant K_d_ = ~1 μM. The subsequent CoMSIA and kNN analyses indicated that mebendazole bears the favorable molecular features that are needed to bind and inhibit TNIK.

Loss of function of the adenomatous polyposis coli (APC), a Wnt signaling pathway inhibitor, and activation mutation of β-catenin are the two major forces driving transformations in colorectal cancers^1^,^2^. However, to date few druggable targets involved in the Wnt pathway have been identified. TRAF2 and NCK-interacting kinase (TNIK) was recently characterized as an essential activator of TCF4/β-catenin transcriptional programme. It is recruited to the promoters of the Wnt target genes and directly phosphorylates TCF4^3^,^4^. This kinase also regulates cytoskeleton rearrangements and stress responses through the Rap2A and c-Jun N-terminal kinase (JNK) pathway, respectively^5^,^6^. Knockdown or mutation of the TNIK kinase domain downregulates the canonical Wnt pathway and JNK pathway, and thus triggers the apoptosis^7^. Since the kinase activity is essential for activation of the β-catenin pathway, TNIK is an attractive therapeutic target against colorectal cancer that obtains aberrant Wnt signaling.

While numerous clinical-relevant kinase inhibitors have been approved^8^,^9^, development of inhibitors targeting TNIK is still in the very early stage. Recently, Yamada *et al*. patented a series of thiazole-4-carboxamide derivatives, including 48 compounds which are able to inhibit TNIK at sub-micromolar concentration (defined as Dataset I)^10^. Similarly, Davis *et al*. profiled the binding affinities of 72 known, chemically diverse kinase inhibitors against 442 kinases, and found a number of compounds exhibited significant binding to TNIK, with dissociation constant ranging from 4.7 nM to 8.5 μM (defined as Dataset II)^11^. However, the biopharmaceutical properties (bioavailability, pharmacokinetics, etc.) of the Dataset I compounds need to be further optimized, and most of the kinase inhibitors in Dataset II are notorious for their known side effects^12^.

Herein, we conducted 2D- and 3D-QSAR studies based on these two datasets and attempted to identify novel TNIK inhibitors with preferred biopharmaceutical properties. With Dataset I, we derived Comparative Molecular Field Analysis (CoMFA), Comparative Molecular Similarity Indices Analysis (CoMSIA), and variable-selection k-nearest neighbor (kNN) models, from which the 3D-molecular fields and 2D-descriptors critical for TNIK inhibitory activity were discovered. Based on Dataset II, a CoMSIA-SIMCA (Soft Independent Modeling by Class Analogy^13^) classification model was obtained and used to screen 1,448 currently marketed drugs. Upon experimental validation using the KINOMEscan platform^14^ (Ambit Biosciences; http://www.kinomescan.com), we found that an anthelmintic drug, mebendazole, could selectively inhibited 91.8% of the TNIK signal at 10 μM, with a dissociation constant (K_d_) of ~1 μM. Mebendazole binds to the ATP-binding pocket of TNIK in a similar fashion as dasatinib, and both of our CoMSIA and kNN models demonstrated that the compound possesses the required molecular pharmacophores and properties to bind TNIK. This study represents a unique ligand-based framework for drug repurposing against a specific protein target critical for colorectal cancer treatment.

## Results

### CoMFA and CoMSIA modeling

**Figure 1:**
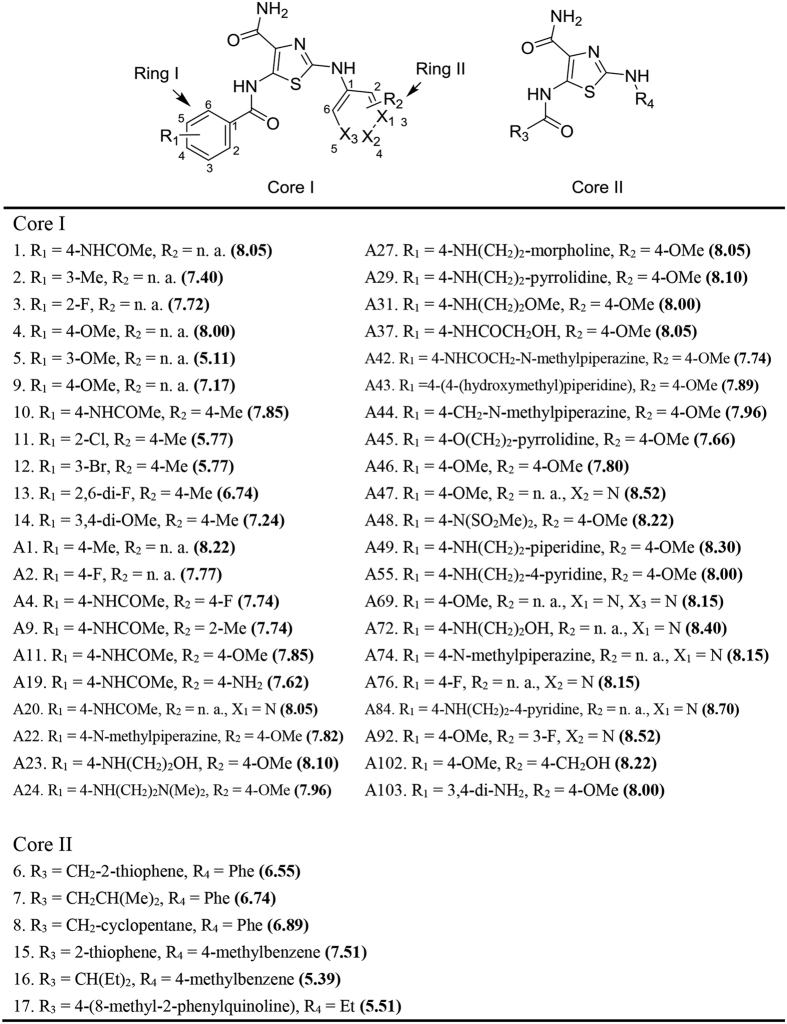
Chemical structures of the thiazole-4-carboxamide derivatives (dataset I). The values in the parentheses are *pIC*_*50*_.

As all compounds in Dataset I are thiazole-4-carboxamide derivatives (Fig. 1), it provides a reliable structural basis to perform ligand alignment for quantitative CoMFA and CoMSIA studies. Using the Sphere Exclusion (SE) algorithm^15^, we divided this dataset into a training set (38 compounds) and a test set (10 compounds). The statistics of the CoMFA and CoMSIA models are shown in Table 1. Compared to the CoMFA model, the CoMSIA model achieved a slightly better leave-one-out cross-validation *q*^2^ (0.685), along with comparable 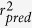 (0.774) and standard error of prediction (0.273). Intriguingly, the hydrophobic term, rather than the steric or electrostatic term, has the largest contribution to the *IC*_*50*_ prediction (Table 1).

**Figure 2:**
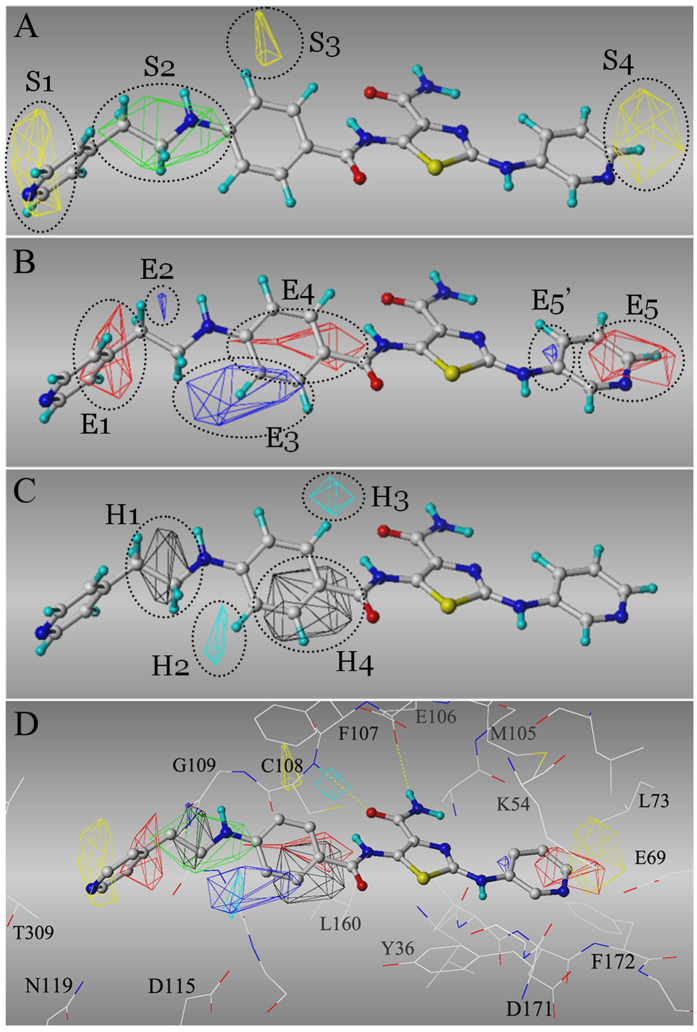
CoMSIA model derived from dataset I. The most active inhibitor, **A84** (in sticks), is used as an example to illustrate the CoMSIA fields (in grids). CoMSIA fields (**A**) Yellow – sterically unfavorable region; Green – sterically favorable region; (**B**) Blue – electronegative unfavorable (or electropositive favorable) region; Red – electronegative favorable (or electropositive unfavorable) region; (**C**) Cyan – hydrophobicity unfavorable region; Black – hydrophobicity favorable region. (**D**) Overlapping the CoMSIA fields to TNIK kinase domain (in lines). Yellow dashed lines indicated the hydrogen bonds with the hinge.

This CoMSIA model also offers structural insights for lead optimization of this thiazole-4-carboxamide series. For example, extension of the *ring I* on the para-position is sterically favorable, as indicated by the *S2* region in Fig. 2A. In contrast, modifications on the ortho- or meta- position, especially the meta-position, are sterically prohibited (*S3* in Fig. 2A). One may notice that the *S3* region is located at the carboxamide side of *ring I*, and compounds such as **5**, **11** and **12**, may flip *ring I* to avoid the sterically unfavorable *S3* region when binding to TNIK. However, compound **17**, which adopts bulky substituents on both sides of *ring I*, has no means to circumvent the *S3* region, and thus it obtains the highest *IC*_*50*_. Furthermore, CoMSIA model suggested that the orth-/meta-position of *ring I* favors single electropositive and hydrophilic group (e.g., amide in compound **103**), and disfavors hydrophobic or electronegative group (e.g., Cl or Br as in compound **11** and **12**), as indicated by *E3*, *H2* and *H3* regions (Fig. 2B,C).

Further analysis of the *S2* and *H1* regions showed that a linear, three-heavy-atom para modification of ring I contributes positively to the *IC*_*50*_ (Fig. 2A,C). The 4^th^ heavy atom favors an electronegative one, such as oxygen (*E1* region in Fig. 2B). For example, compared with compound **A11**, the addition of a hydroxyl group to the 4^th^ heavy atom (compound **A37**) lowers *IC*_*50*_. For the distal modifications of ring I, the sterically unfavorable *S1* region restricts the maximum length of the substituent. To achieve the best TNIK inhibition, the estimated ideal length should range from 5.0 Å to 7.0 Å (Fig. 2A). However, it is worth noting that the contributions of *S1*, *E1* and *H1* regions are usually below 0.3 *pIC*_*50*_ units, significantly less than other regions (Supplementary Table 2 for comparisons).

On the other side of the core, we observed that a small electronegative substituent on the para-position is energetically favorable on *ring II*, as indicated by *S4* and *E5* in Fig. 2A,B. This finding is consistent to *IC*_*50*_ values that follow the ascendant trend: 4-pyridine (X_3_ = N) < 3-pyridine or 5-pyrimidine (X_1_ or/and X_3_ = N) < Phe (R_2_ = H) < Phe-4-OMe (R_2_ = OMe) (Supplementary Table 2). Accordingly, the existence of electronegative-favorable *E5* region could result in the electropositive-favorable *E5’* region.

### Molecular docking confirmed CoMSIA model

As we analyzed, no compound in Dataset I bears the pharmacophore of the allosteric inhibitors of TNIK (type 2 kinase inhibitor^16^) (data not shown). Indeed, this chemical series most likely functions as type 1 kinase inhibitors^16^, which bind the ATP-binding pocket without flanking to the allosteric site. The molecular docking confirmed this hypothesis, because the thiazole-4-carboxamide core was consistently docked to the adenine site in three difference receptor models, including TNIK^close^, TNIK^open^ and TNIK^DFG-out^. Also the ligand binding mode is in agreement with the aforementioned CoMSIA model. Figure 2D demonstrated the predicted binding mode for compound **A84** in TNIK^close^. Based on the Traxler model which breaks the ATP-binding pocket into five subcomponents^17^, the adenine site is occupied by thiazole-4-carboxamide forming the hydrogen bonds to the hinge (E106 and C108 in Fig. 2D). Ring I is located at the hydrophobic pocket I, while ring II is buried in the hydrophobic pocket II and close to the gatekeeper residue (M105).

In particular, the meta-carbon of the ring I (corresponding to the *E3* and *H2* regions) is only 4.4 Å away from the carboxylatic oxygen of D115 (Fig. 2D). Moreover, the sterically-unfavorable *S3* and hydrophobicity-unfavorable *H3* regions are located in the hydrophobic pocket I. These observations buttressed the CoMSIA model that a small, electropositive and hydrophilic, meta- substituent of ring I is structurally preferred. Surprisingly, the electropositively favorable *E2* region forms a polar contact with the carbonyl group of G109 (Fig. 2D). Finally, due to the bulky gatekeeper residue M105, the small electronegative modification(s) of ring II is apparently more energetically favorable than the large hydrogen-rich ones. This is consistent with the above analysis of the *S4* and *E5* regions (Fig. 2A,B).

### Ligand-based kNN modeling

**Figure 3:**
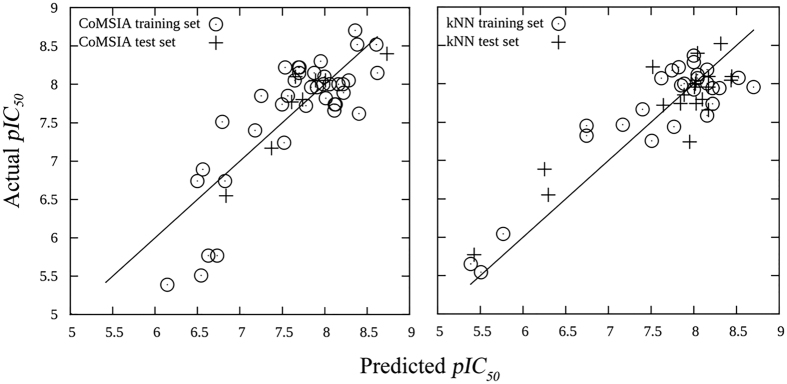
Predicted *pIC*_*50*_ values versus actual *pIC*_*50*_ values for CoMSIA model (left) and kNN model (right). The predicted *pIC*_*50*_ for both training sets are predicted by leave-one-out cross-validation.

The best six kNN models are shown in Table 2. All of these models had less than five descriptors, and both leave-one-out cross-validated training set *q*^*2*^ and testing set *r*^*2*^ were over 0.74, indicating the high predictive abilities of the resultant models. Of note, the kNN model #1, which contains 27 compounds in the training set and 21 in the test set, was able to accurately predict the *pIC*_*50*_ values with *q*^*2*^ = 0.81 and *r*^*2*^ = 0.78 using only three descriptors. The right plot in Fig. 3 demonstrated that our variable selection method particularly determined four types of descriptors that contribute mostly to the kNN models: KierA2 (second alpha modified shape index), diameter/radius, GCUT_SLogP/BCUT_SLogP, and PEOE_VSA_HYD (total hydrophobic van der Waals surface area).

Intriguingly, we discovered that there are inverse correlations between *pIC*_*50*_ with KierA2 and PEOE_VSA_HYD for molecules with a radius below 7 (Supplementary Fig. 1). The correlation coefficients for *pIC*_*50*_ with KierA2 and PEOE_VSA_HYD are 0.78 and 0.55, respectively. KierA2 represents the overall shape of the molecule. Generally, a linear compound has alower KierA2 than a complex one. PEOE_VSA_HYD describes the total hydrophobic van der Waals surface area, in which the partial charges are computed with the partial equalization of orbital electronegatives. These two inverse correlations implied that the core scaffold of TNIK inhibitors (e.g., radius < 7) should be linear and hydrophilic in order to maximize the efficiency of design. This is consistent to the observation that the steric bulky or hydrophobic unfavorable regions, such as *S3*, *S4*, *H2* and *H4*, are located at the hydrophobic pockets I and II (Fig. 2D).

### Accurate categorization of TNIK-binding kinase inhibitors by CoMSIA-SIMCA

While the 2D- and 3D-QSAR of Dataset I have provided essential structural guidance for computer-aided design of TNIK inhibitors, additional studies of Dataset II, which covers much more chemical spaces, can expand the applicability domain of our models. To this end, we conducted SIMCA^13^ analysis with Dataset II. SIMCA is a widely-used classification algorithm that develops predictive models using principal component analysis (PCA) based on the precategorized training set. Due to the structural diversity of Dataset II, we filtered out all type 2 kinase inhibitors and allosteric inhibitors, leading to 54 type 1 kinase inhibitors (Supplementary Table 1). These compounds were then grouped into four categories. According to their respective *pK*_*d*_ values, we intentionally name them as *Category IV*: *pK*_*d*_ < 5; *Category V*: 5 ≤ *pK*_*d*_ < 6; *Category VI*: 6 ≤ *pK*_*d*_ < 7; *Category VII*: *pK*_*d*_ ≥ 7. We also divided the dataset into a training set (48 compounds) and a test set (6 compounds) for model building (Supplementary Table 5). The CoMSIA fields were computed based on the receptor-guided alignment using rigorously-designed molecular dockings with respect to the available structural data (see Materials and Methods for details).

As indicated by the confusion matrix (Table 3), we derived a robust CoMSIA-SIMCA model in which 91.7% of kinase inhibitors were accurately classified (cross-validated) for the training set, while only four compounds were misclassified after five-group cross-validation. The predictive ability of this model was further validated on the test set, in which only one compound (AZD-2171) was misclassified. It is worth noting that three (out of five) misclassified compounds, including SB-203580 (*pK*_*d*_ = 6.086), TAE-684 (*pK*_*d*_ = 5.921) and AZD-2171 (*pK*_*d*_ = 6.092), have *pK*_*d*_ values close to the classification margin. As expected, these three compounds were classified to the adjacent categories: SB-203580 - *Category V*, TAE-684 - *Category VI* and AZD-2171 - *Category V*. Furthermore, the distances between categories followed the trend of binding affinities (Table 4), indicating that the SIMCA model can semi-quantitatively reflect the strength of TNIK binding. The details of the categorization results are available in Supplementary Table 5. To exclude the possibility of overfitting, we also conducted Y-randomization as we described previously^18^,^19^,^20^,^21^. It failed to accurately predict the activity (data not shown), demonstrating the robustness of our models.

### Identification of mebendazole as TNIK inhibitor

Herein, we have derived predictive CoMSIA-SIMCA models that can be utilized to efficiently screen a drug-like database to discover novel TNIK inhibitors. The structural insights provided by our modeling can further improve the virtual screening accuracy. To this end, we screened 1,448 US Food and Drug Administration (FDA)-approved small-molecule drugs, aiming to identify potential TNIK inhibitor from the marketed drugs that have desired absorption, distribution, metabolism, and elimination (ADME) properties and already passed the strict safety/toxicity investigations. Not surprisingly, the CoMSIA-SIMCA model identified three kinase inhibitors, sunitinib, gefitinib and dasatinib, which could block TNIK activities. Such predictions are in agreement with some experimental reports: Sunitinib was classified in *Category VII* (actual *K*_*d*_ = 25 nM), whereas gefitinib was classified in *Category V* (actual *K*_*d*_ = 6.9 μM) (Table 5).

**Figure 4:**
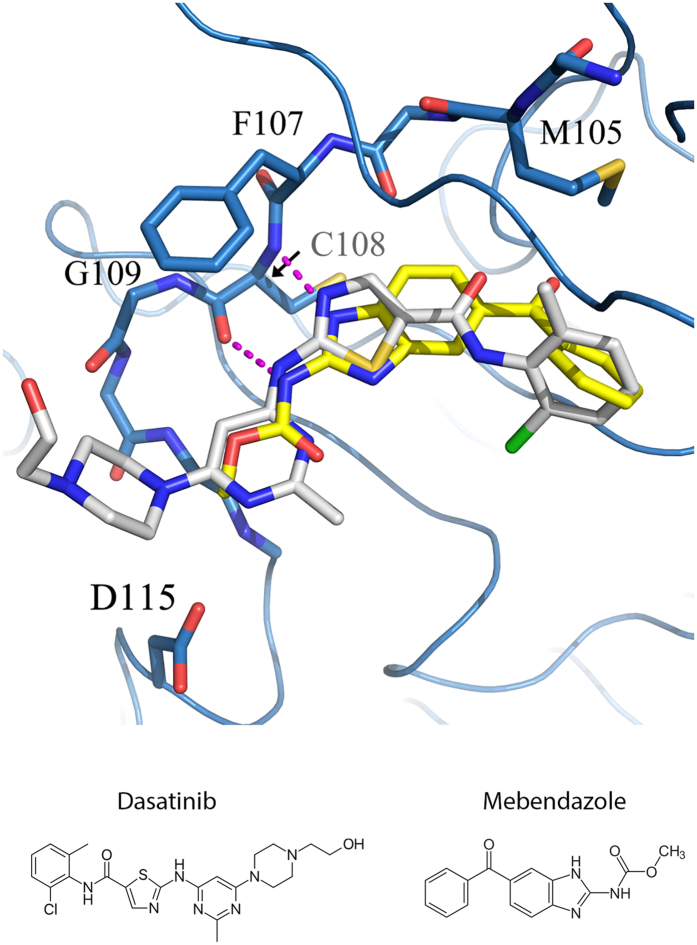
The binding modes of dasatinib (white) and Mebendazole (yellow) in TNIK kinase domain. The blue ribbons represents TNIK kinase domain, and the hinge residues and D115 side chain are highlighted with sticks. The magenta dashed lines represent the hydrogen bonds between Mebendazole and hinge. Chemical structures of Dasatinib and Mebendazole are also shown.

For the first time, mebendazole (MBZ), which was originally designed as an anthelmintic drug^22^, was identified to be a TNIK inhibitor. The CoMSIA-SIMCA model classified MBZ as a potent agent (*Category V* - *K*_*d*_ ranging from 1 μM to 10 μM). Our follow-up molecular docking indicates that MBZ binds to the adenine site of TNIK. The 3-N of benzimidazole forms hydrogen bonding interactions with the C108 amide, whereas the carbamate group interacts with C108 via its carbonyl group. This hydrogen bonding pattern of MBZ binding to TNIK is similar to that of dasatinib (Fig. 4). When referred to the CoMSIA fields shown in Fig. 2A–D, both dasatinib and MBZ circumvent the unfavorable bulky *S3* region and minimize the hydrophobicity in the *H2* region. However, MBZ is even less bulky around the hydrophobic pocket II (corresponding to *S4* region) than dasatinib. Conforming to our kNN modeling, MBZ has the desired molecular properties as a TNIK inhibitor: Its radius was 7, whereas the KierA2 (5.24) and PEOE_VSA_HYD (213.47) were significantly below the averages of KierA2 (8.03) and PEOE_VSA_HYD (297.95) obtained from Dataset I.

### Experimental validation of MBZ as a TNIK inhibitor

We performed experimental studies of MBZ using the KINOMEscan platform to evaluate its binding to TNIK and selectivity among different kinases. Our result shows that MBZ inhibits 91.8% of TNIK activity at 10 μM. Compared with other inhibitors, such as dasatinib (89% inhibition at 10 μM) and gefitinib (66% inhibition at 10 μM), the *K*_*d*_ of MBZ binding to TNIK is likely to be ~1.0 μM (Table 5). In contrast, MBZ does not exhibit significant inhibitory effect, defined as over 70% inhibition of the control, on ABL2, EGFR, MEK1, PDPK1, PIK3CA, and ACK1 at 10 μM. Since TNIK acts as an activator of the Wnt/β-catenin/TCF4 pathway^3^,^4^, this finding is consistent to some previous study showing that MBZ exhibits a potent cytotoxicity against β-catenin-active colon and non-small-cell lung cancer cell lines^23^.

## Discussion

In the present study, we conducted comprehensive modeling studies of TNIK inhibitors and developed CoMFA/CoMSIA, kNN, and CoMSIA-SIMCA models, which were then rationally applied to screen and identify an approved drug, mebendazole, as a potent TINIK inhibitor for cancer therapy. In particular, CoMSIA and kNN model provided valuable structural insights that the TNIK inhibitors usually favor the linear and hydrophilic moieties rather than the complex and hydrophobic groups. Meanwhile, CoMSIA-SIMCA classification model provided a platform to conduct efficient primary screening of potential TNIK inhibitors. As part of the recent polypharmacology and drug repurposing efforts^24^,^25^ such comprehensive studies and the resultant models are ready to be used for compound repositioning against other targets.

Mebendazole (MBZ), as a marketed drug, is suited for further clinical study considering its promising safety profile^22^. Several studies have suggested MBZ as a potent antitumor agent. For instance, MBZ was demonstrated significant inhibition against the growth and metastasis of the adrenocortical carcinoma, both *in vitro* and *in vivo*^26^. In addition, studies have shown that MBZ could cause a mitotic arrest and a time-dependent apoptotic response in various cancercell lines^27^,^28^. However, the mechanism of its antitumor property remains elusive. The most well-known target of MBZ is tubulin, in which MBZ directly binds tubulin and impairs the tubulin polymerization^27^,^29^.

Herein, we identified a novel mechanism of action for MBZ which targets an oncogenic protein, TNIK, in a clinical-relevant signaling pathway, particular in colorectal cancer. TNIK phosphorylates S154 of TCF4, and its catalytic activity has proved to be essential for the colorectal cancer growth^3^. According to our experimental testing results, the *K*_*d*_ for MBZ against TNIK kinase domain is around 1 μM, which is of the same order of magnitude as the reported *K*_*d*_ value to mammalian brain tubulin (0.5 μM)^30^. In addition, mebendazole is a FDA-approved drug and can be used at a significantly higher dosage (up to 200 mg/kg with daily use^31^) without severe side effects. In addition, mebendazole may exhibit a synergic anti-tumor effect with other anti-cancer drugs through disrupting cytoskeleton because TNIK kinase activity has proven essential to regulate the F-actin fiber formation^32^. Such advantages render the possibility of quickly translating the discovery into clinical setting for cancer treatment in the near future.

## Materials and Methods

### Dataset

We collected a series of potent TNIK inhibitors from a previous study, including 48 patented TNIK inhibitors with known *IC*_*50*_ values (Dataset I)^10^, and 72 kinase inhibitors with known dissociation constant values (*K*_*d*_) (Dataset II)^11^. All of the patented compounds along with the inhibitory IC_50_ values are available in Fig. 1. The chemical structures and the *K*_*d*_ values were retrieved from the ChEMBL database (ID: 1908790). The kinase inhibitors in Dataset II were classified into type 1, type 2 and allosteric inhibitors based on the available literatures and protein-ligand complex structures (Supplementary Table 1). –log(*IC*_*50*_) and –log(*K*_*d*_) values were computed as the dependent variable for all analyses. Since Dataset I and II cover different chemical space, they are curated herein to achieve different goals and thus we kept them separate during model building.

### 3D-QSAR by CoMFA and CoMSIA

3D-molecular alignment were prepared using SYBYL 8.1 (Tripos, Ltd, US) and Molecular Operating Environment (MOE 2010.10). For dataset I, molecular alignments were performed using substructure overlap thiazole-4-carboxamide core with respect to the docking poses (see molecular docking for details). The resulting alignment was refined by flexible alignment using MOE. The alignment is available from the authors upon request. CoMFA and CoMSIA were performed by SYBYL 8.1 using the default parameter (Tripos standard field, 2 Å grid spacing, dielectric distance 1/*r*^2^, 30 kcal/mol cutoff) probed by an *sp*^3^ carbon with a charge of +1. CoMFA steric energy (Lennard-Jones) and electrostatic (Coulomb) energy were calculated. CoMSIA steric, electrostatic and hydrophobic energies were calculated with the attenuation factor = 0.3. Pullman charges were used for electrostatic field calculations. 3D-QSAR was performed by partial least squares (PLS) analyses. Dataset I was divided into a training set (38 compounds) and a test set (10 compounds) based on the Sphere Exclusion (SE) algorithm^15^. The details of data splitting are available in Supplementary Table 3. Leave-one-out cross-validation was used for the training set. The predictive ability the non-cross-validated models were validated using the test set. The statistics of the resulted CoMFA and CoMSIA models are available in Table 1.

### 2D-QSAR by variable-selected kNN

2D-QSAR was performed on dataset I. A total of 186 2D-descriptors were calculated with MOE 2010.10. The resulting descriptors were normalized to [0, 1], and the descriptors with zero standard deviation were removed. As described previously, dataset splitting was performed using an SE8 program implemented with sphere exclusion algorithm^15^. The details of data splitting are available in Supplementary Table 4. The resulting 50 training and test sets which consider the diversity of descriptors were used to build the predictive k-nearest neighbor (kNN) models. The implementation of this variable-selection kNN algorithm has been published elsewhere^33^. Concisely, the simulated annealing algorithm was employed to explore the entire descriptor spaces and optimize the descriptor subset based upon the leave-one-out (LOO) cross-validation *q*^*2*^. The models whose *q*^*2*^s satisfied the cutoff were validated on their respective test sets (non-cross-validated). Only the kNN models satisfying the criteria that “*q*^*2*^ > 0.7 and *r*^*2*^ > 0.7 and number of descriptors <10” were kept for further analysis.

### Molecular docking

High-resolution crystal structure of TNIK in complex with Wee1 kinase inhibitor was obtained from Protein Data Bank (PDB ID: 2X7F). Due to the structural defects in the ATP binding site, we aligned all available five chains in 2X7F, and chain B was used as receptor because its ATP-binding site had the least number of missing residues (e.g., E29, T35, K41, K54, M56, E69, K155, E163). The missing ζ-amine of K54 and K155 were manually added, and these two ζ-amine moieties were subjected to energy minimization optimization. Considering the dynamics of the TNIK kinase domain, we established three different models, TNIK^open^, TNIK^close^ and TNIK^DFG-out^, to represent the lobe-open, lobe-close and DFG-out conformations. TNIK^close^ used the original coordinates in 2X7F. Based on TNIK^close^, we built TNIK^open^ by modeling the TNIK lobe (residue 29–41) with respect to a template in which lobe is in open conformation (PDB ID: 3NIZ, residue 27–39). Similarly, TNIK^DFG-out^ was built by modeling the TNIK activation loop (residue 170–173) with respect to the sorafenib-bound template (PDB ID: 3HEG^34^, residue 167–170). GOLD 5.1 (CCDC)^35^ was used for molecular docking. Based on the expert knowledges on kinase and the crystal structure of p38α in complex with an isothiazole-4-carboxamdide derivative, CP-547632 (PDB ID: 3L8S)^36^, we constrained the hydrogen bonds with the carbonyl group of M106 localized at the hinge. All of the docking parameters were the same as previously described^37^, except that ChemScore-Kinase scoring function was used because its ability to recognize the activated CH groups (e.g., the CH group next to the nitrogen in a pyridine) for H-bonds. In addition, the soft potentials on Y36 were employed for TNIK^close^ to account for the flexibility of the tip of the lobe.

### CoMSIA-SIMCA

A total of 54 type 1 kinase inhibitors were selected from Dataset II for CoMSIA-SIMCA modeling (Supplementary Table 1). The selected molecules were aligned by molecular docking to TNIK^open^ and TNIK^close^. For each molecule, only the top-ranking pose that was similar to the one crystallized in the reference structures (root mean square deviation <3.0 Å and same H-bond pattern to the hinge) is kept for CoMSIA-SIMCA modeling. The CoMSIA fields (steric, electrostatic, hydrophobic, H-bond acceptor, H-bond donor) were computed using the parameters described above. These type 1 inhibitors were classified into four groups based on their respective *pK*_*d*_ values (Table 3), and the dataset was divided into a training set (48 compounds) and a test set (6 compounds). The SIMCA model derived from the training set was validated by five-group cross-validation. The details of data splitting and predictions are available in Supplementary Table 5.

### Experimental validation

We collected 1,448 FDA-approved small-molecule drugs from the DrugBank^38^. These molecules were docked to TNIK^open^ and TNIK^close^ using the same procedure described above, and the binding affinities to TNIK were predicted by CoMSIA-SIMCA model. Ten hits (Amodiaquine, Gefitinib, Sunitinib, Mebendazole, Dasatinib, Lapatinib, Imatinib, Sorafenib, Flavoxate, and Flavopiridol) that were predicted to be *Category V*, *VI or VII* were selected for experimental validation using KINOMEscan (DiscovRx, San Diego, CA) against seven kinases (TNIK, Activated CDC42 kinase 1 (ACK1), phosphatidylinositol–4,5-bisphosphate 3-kinase catalytic subunit alpha (PIK3CA), 3-phosphoinositide dependent protein kinase-1 (PDPK1), Abelson tyrosine-protein kinase 2 (ABL2), Epidermal growth factor receptor (EGFR), Mitogen-activated protein kinase kinase (MEK)). Briefly, this KINOMEscan platform quantifies the amount of DNA-tagged kinase that is unable to bind the immobilized reference ligand after adding the testing ligand by qPCR. The selected compounds were tested at 0.1 μM and 10 μM. The results of KINOMEscan testing were reported as the percentage of the control (% Ctrl) where lower values suggest stronger hits: % Ctrl = (test compound signal − positive control signal)/(negative control signal − positive control signal).

## Additional Information

**How to cite this article**: Tan, Z. *et al*. Comprehesnive Modeling and Discovery of Mebendazole as a Novel TRAF2- and NCK-interacting Kinase Inhibitor. *Sci. Rep.* **6**, 33534; doi: 10.1038/srep33534 (2016).

## Supplementary Material

Supplementary Information

## Figures and Tables

**Table 1:** Summary of CoMFA and CoMSIA models.

Statistics	CoMFA	CoMSIA
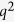 ^a^	0.622	0.685
SEP^b^	0.586	0.534
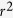 ^c^	0.987	0.992
SEE^d^	0.108	0.084
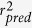 ^e^	0.779	0.774
SEP_pred_^f^	0.263	0.273
Components^g^	10	10
*F*^h^	216.204	353.298
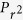 ^i^	0.000	0.000
Fraction		
Steric	0.573	0.215
Electrostatic	0.427	0.330
Hydrophobic	NA^j^	0.455

^a^LOO cross-validated correlation coefficient (training set).

^b^LOO cross-validated standard error of prediction (training set).

^c^Non-cross-validated correlation coefficient (training set).

^d^Standard error of estimate (training set).

^e^Correlation coefficient for the test set.

^f^Standard error of prediction for the test set.

^g^Optimal number of components.

^h^F-test value.

^i^Probability of obtaining the F value by chance.

^j^Hydrophobic contribution not available in CoMFA.

**Table 2:** Summary of kNN models.

Models	Data splitting (training/testing)	Neighbors^a^	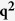 ^**b**^	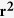 ^**c**^	Descriptors
1	27/21	2	0.81	0.78	KierA2, GCUT_SLogP_0, radius
2	28/20	2	0.82	0.74	KierA2, BCUT_SLogP_3, diameter
3	33/15	2	0.75	0.83	PEOE_VSA_HYD, vsa_acc, radius
4	35/13	2	0.77	0.86	PEOE_VSA_HYD, SMR_VSA6, PEOE_PC+, radius
5	36/12	2	0.74	0.89	KierA2, b_ar, radius
6	37/11	4	0.76	0.93	KierA2, GCUT_SLogP_0, radius

^a^Optimal number of nearest neighbors.

^b^LOO cross-validated correlation coefficient for the training set.

^c^Correlation coefficient for the test set.

**Table 3:** CoMSIA-SIMCA analysis for the training set upon five-group cross-validation.

Actual/Predicted	IV	V	VI	VII	Total
IV	21	1	0	0	22
V	0	8	1	0	9
VI	0	1	9	0	10
VII	0	0	1	6	7

*Category IV*: *pKd* < 5; *Category V*: 5 ≤ *pKd* < 6; *Category VI*: 6 ≤ *pKd* < 7; *Category VII*: *pKd* ≥ 7.

**Table 4:** Distance between categories obtained from CoMSIA-SIMCA model.

	ActualCat4	ActualCat5	ActualCat6	ActualCat7
ProjectedCat4	201.395	270.533	320.406	381.345
ProjectedCat5	262.338	165.989	241.476	292.651
ProjectedCat6	305.968	231.206	181.780	244.579
ProjectedCat7	377.213	277.938	239.415	170.452

**Table 5:** The experimental results from KINOMEscan scanELECT.

Compound	Kinase	% Ctrl		*K*_*d*_ (μM)
		0.1 μM	10 μM	
Sunitinib	TNIK	37	0	0.025
Dasatinib	TNIK	82	11	2.0
Gefitinib	TNIK	100	34	6.9
Lapatinib	TNIK	90	93	>10
Flavopiridol	TNIK	92	33	>10
**Mebendazole**	**TNIK**	**97**	**8**.**2**	ND
	ABL2	100	33	ND
	MEK1	100	38	ND
	EGFR	100	83	ND
	ACK1	100	98	ND
	PDPK1	100	100	ND
	PIK3CA	100	100	ND

The values were reported as percentage of control, where lower value indicates stronger binding. ND – not determined.
