# Supplementary material for: Comprehensive Modeling and Discovery of Mebendazole as a Novel TRAF2- and NCK-interacting Kinase Inhibitor
Source: Sci Rep. 2016 Sep 21;6:33534. doi: 10.1038/srep33534 (PMC5030704; doi:10.1038/srep33534)

Discovery of Mebendazole as a novel TRAF2- and NCK-interacting kinase inhibitor

Zhi Tan[1,2,*], Lu Chen[1,2,*], Shuxing Zhang [1,2]

[1]Integrated Molecular Discovery Laboratory, Department of Experimental Therapeutics, The University of Texas MD Anderson Cancer Center, Houston, TX 77030
[2]The University of Texas Graduate School of Biomedical Sciences, Houston, TX 77030, USA.

[*]These authors contributed equally to this work.
Correspondence and requests for materials should be addressed to Shuxing Zhang, Ph.D.
Tel: +1-(713)745-2958
Fax: +1-(713)794-5577
Email: shuzhang@mdanderson.org

Supplementary Table 1. 72 kinase inhibitors and their type of binding mode. We used K_d = 100 μM to indicate the inactive compounds. The actives are shaded in yellow, and the inactives are shaded in blue.

Compound	Binding Mode	Alternative Name	K_d to TNIK (μM)	pK_d to TNIK	PDB ID of reference structure
A-674563	Type I		0.1400	6.854	2UZT, 2UZW
AB-1010	Type II	Masitinib	100.0000	4.000	
ABT-869	Type II	Linifanib	2.8000	5.553	
AC220	Type II	Quizartinib	2.8000	5.553	
AG-013736	Type I	Axitinib	0.1800	6.745	4AG8
AMG-706	Type II	Motesanib	100.0000	4.000	3EFL
AST-487	Type II		0.0690	7.161	3UGC (similar)
AT-7519	Type I		100.0000	4.000	2VU3
AZD-1152HQPA	Type II		8.5000	5.071	
AZD-2171	Type I	Cediranib	0.8100	6.092	
AZD-6244/ARRY-886	Allosteric	Selumetinib	100.0000	4.000	
BI-2536	Type I		100.0000	4.000	2RKU
BIBF-1120 (derivative)	Type I		0.0530	7.276	3C7Q
BIBW-2992	Type I	Afatinib	100.0000	4.000	4G5J
BIRB-796	Type II	Doramapimod	0.1400	6.854	1KV2
BMS-345541	Type I		100.0000	4.000	
BMS-387032/SNS-032	Type I		100.0000	4.000	
BMS-540215	Type I	Brivanib	0.1600	6.796	
CEP-701	Type I	Lestaurtinib	0.0054	8.268	1R0P (similar)
CHIR-258/TKI-258	Type I	Dovitinib	0.0240	7.620	
CHIR-265/RAF-265	Type II		1.5000	5.824	
CI-1033	Type I	Canertinib	8.2000	5.086	2VRX (similar)
CI-1040	Allosteric		100.0000	4.000	3EQC
CP-690550	Type I	Tofacitinib	100.0000	4.000	3LXK
Crizotinib	Type I		100.0000	4.000	2YFX
Dasatinib	Type I		2.0000	5.699	2Y6O, 3G5D
Erlotinib	Type I		4.5000	5.347	1M17
EXEL-2880/GSK1363089	Type II	Foretinib	0.2100	6.678	3LQ8
Flavopiridol	Type I	Alvocidib	100.0000	4.000	3BLR
GDC-0879	Type I		2.3000	5.638	3D4Q (similar)
GDC-0941	Type I		100.0000	4.000	2WXP
Gefitinib	Type I		6.9000	5.161	3UG2
GSK-1838705A	Type I		100.0000	4.000	3EKK (similar)
GSK-461364A	Type I		100.0000	4.000	2XNM (similar)
GSK-690693	Type I		100.0000	4.000	3D0E
GW-2580	Type II		100.0000	4.000	4AT5

HKI-272	Type I	Neratinib	0.1400	6.854	3W2Q
Imatinib	Type II		100.0000	4.000	2HYY, 2PL0
INCB18424	Type I	Ruxolitinib	100.0000	4.000	
JNJ-28312141	Type I		0.1500	6.824	3KRJ
Ki-20227	Type II		2.3000	5.638	2RL2 (similar)
KW-2449	Type I		0.0280	7.553	
Lapatinib	Type I		100.0000	4.000	3BBT, 1XKK
LY-317615	Type I	Enzastaurin	100.0000	4.000	1UU7 (similar)
LY-333531	Type I	Ruboxistaurin	100.0000	4.000	1UU3
MLN-120B	Type I		100.0000	4.000	
MLN-518	Type II	Tandutinib	100.0000	4.000	
MLN-8054	Type I		100.0000	4.000	2X81
Nilotinib	Type II		100.0000	4.000	3GP0
Pazopanib	Type I		0.3100	6.509	3CJG (similar)
PD-173955	Type I		0.4300	6.367	1M52
PHA-665752	Type I		0.3100	6.509	2WKM
PI-103	Type I		100.0000	4.000	2X6K
PKC-412	Type I	Midostaurin	1.6000	5.796	1AQ1 (similar)
PLX-4720	Type I		2.5000	5.602	3C4C
PP-242	Type I		0.2700	6.569	3T3V (similar)
PTK-787	undetermined	Vatalanib	100.0000	4.000	
R406	Type I	Tamatinib	1.3000	5.886	3FQS
R547	Type I		100.0000	4.000	2FVD
SB-203580	Type I		0.8200	6.086	3ZS5
SGX-523	Type I		100.0000	4.000	3DKG
SKI-606	Type I	Bosutinib	0.0310	7.509	3UE4
Sorafenib	Type II		7.9000	5.102	3HEG
Staurosporine	Type I		0.0047	8.328	4ERW
SU-14813	Type I		0.0900	7.046	3G0E (similar)
Sunitinib	Type I		0.0250	7.602	3G0E
TAE-684	Type I		1.2000	5.921	2XB7
TG-100-115	Type I		100.0000	4.000	
TG-101348	Type I		100.0000	4.000	
Vandetanib	Type I		2.3000	5.638	
VX-680/MK-0457	Type I	Tozasertib	1.8000	5.745	3E5A, 2XYN
VX-745	Type I		100.0000	4.000	3ZSI

Supplementary Table 2. Structural comparison indicated by CoMSIA fields.

Field(s)	Cmpd ID (pIC50)	Changes
S4 & E5	4 (**8.00**) → A46 (**7.80**)	4-H → 4-OMe
	A47 (**8.52**) → 4 (**8.00**)	4-pyridine → Phe
	A47 (**8.52**) → A69 (**8.15**)	4-pyridine → 5-pyrimidine
	1 (**8.05**) → 10 (**7.85**)	4-H → 4-Me
	1 (**8.05**) → A11 (**7.85**)	4-H → 4-OMe
	A11 (**7.85**) → A12 (**7.62**)	4-OMe → 4-NH_2
	A84 (**8.70**) → A55 (**8.00**)	3-pyridine → Phe-4-OMe
	A72 (**8.40**) → A23 (**8.00**)	3-pyridine → Phe-4-OMe
	A74 (**8.15**) → A22 (**7.82**)	3-pyridine → Phe-4-OMe
S3, E3, H2 & H3	9 (**7.17**) → 11 (**5.77**)	2-F → 2-Cl
	9 (**7.17**) → 13 (**6.74**)	2-F → 2,6-diF
	9 (**7.17**) → 12 (**5.77**)	2-F → 3-Br
	A103 (**8.00**) → 14 (**7.24**)	3,4-di-NH_2 → 3,4-di-OMe
	12 (**5.77**) → 5 (**5.11**)	3-Br → 3-OMe
S2 & H1	A23 (**8.10**) → A37 (**8.05**)	4-$NHCH_2CH_2OH$ → 4-$NHCOCH_2OH$
	A47 (**8.52**) → A76 (**8.15**)	4-OMe → 4-F
	A27 (**8.05**) → A42 (**7.74**)	4-NH$(CH_2)_2$-morpholine → 4-$NHCOCH_2$-N-methylpiperazine
E1	A37 (**8.05**) → A11 (**7.85**)	4-$NHCOCH_2OH$ → 4-$NHCOCH_3$
	A23 (**8.10**) → A24 (**7.96**)	4-NH$(CH_2)_2$OH → 4-NH$(CH_2)_2N(CH_3)_2$
E4 & H4	15 (**7.51**) → 6 (**6.55**)	2-thiophene → CH_2-2-thiophene
	15 (**7.51**) → 16 (**5.39**)	2-thiophene → $CH(Et)_2$
S1	A49 (**8.30**) → A27 (**8.05**)	4-NH$(CH_2)_2$-piperidine → 4-NH$(CH_2)_2$-morpholine
	A49 (**8.30**) → A55 (**8.00**)	4-NH$(CH_2)_2$-piperidine → 4-NH$(CH_2)_2$-4-pyridine
	A49 (**8.30**) → A42 (**7.74**)	4-NH$(CH_2)_2$-piperidine → 4-$NHCOCH_2$-N-methylpiperazine

Supplementary Table 3. Details of the data splitting and predictions for the CoMSIA model. The predicted pIC_{50}s for the training set are the LOO cross-validated values.

Training set

ID	Predicted pIC_{50}	Actual pIC_{50}	Predicted – Actual pIC_{50}
2	7.178	7.400	-0.222
3	7.778	7.720	0.058
4	7.985	8.000	-0.015
7	6.824	6.740	0.084
8	6.564	6.890	-0.326
10	7.251	7.850	-0.599
11	6.628	5.770	0.858
12	6.733	5.770	0.963
13	6.500	6.740	-0.240
14	7.522	7.240	0.282
15	6.793	7.510	-0.717
16	6.147	5.390	0.757
17	6.545	5.510	1.035
A1	7.703	8.220	-0.517
A4	8.118	7.740	0.378
A9	8.129	7.740	0.389
A11	7.567	7.850	-0.283
A19	8.405	7.620	0.785
A22	8.013	7.820	0.193
A23	7.996	8.100	-0.104
A24	7.909	7.960	-0.051
A27	8.281	8.050	0.231
A31	8.057	8.000	0.057
A37	7.646	8.050	-0.404
A42	7.496	7.740	-0.244
A43	8.224	7.890	0.334
A44	7.838	7.960	-0.122
A45	8.114	7.660	0.454
A47	8.610	8.520	0.090
A48	7.689	8.220	-0.531
A49	7.953	8.300	-0.347
A55	8.214	8.000	0.214
A69	8.619	8.150	0.469
A74	7.700	8.150	-0.450
A76	7.877	8.150	-0.273
A84	8.360	8.700	-0.340
A92	8.382	8.520	-0.138
A102	7.532	8.220	-0.688
A103	8.162	8.000	0.162

Test set

ID	Predicted pIC_{50}	Actual pIC_{50}	Predicted – Actual pIC_{50}
1	8.010	8.050	-0.040
5	7.977	5.110	2.867
6	6.837	6.550	0.287
9	7.374	7.170	0.204
A2	7.608	7.770	-0.162
A20	7.891	8.050	-0.159
A29	7.653	8.100	-0.447
A46	7.738	7.800	-0.062
A72	8.732	8.400	0.332

Supplementary Table 4. Details of the data splitting and predictions for kNN model 1. The predicted pIC_{50}s for the training set are the LOO cross-validated values.

Training set

ID	Predicted pIC_{50}	Actual pIC_{50}	Predicted – Actual pIC_{50}
2	7.398	7.669	-0.271
4	8.000	8.285	-0.285
7	6.745	7.453	-0.709
9	7.167	7.468	-0.301
11	5.770	6.045	-0.275
13	6.745	7.323	-0.578
15	7.509	7.258	0.250
16	5.387	5.650	-0.262
17	5.509	5.545	-0.037
A2	7.770	7.438	0.332
A11	7.854	7.981	-0.127
A19	7.620	8.075	-0.455
A20	8.046	8.072	-0.027
A22	7.824	8.218	-0.394
A27	8.046	8.116	-0.070
A31	8.000	8.369	-0.369
A42	7.745	8.173	-0.429
A43	7.886	8.000	-0.114
A47	8.523	8.077	0.445
A48	8.222	7.945	0.277
A49	8.301	7.945	0.356
A55	8.000	7.925	0.075
A69	8.155	8.185	-0.030
A74	8.155	8.005	0.150
A76	8.155	7.586	0.569
A84	8.699	7.956	0.743
A102	8.222	7.737	0.485

Test set

ID	Predicted pIC_{50}	Actual pIC_{50}	Predicted – Actual pIC_{50}
1	8.030	8.046	-0.016
3	7.646	7.721	-0.075
5	6.129	5.114	1.015
6	6.297	6.553	-0.256
8	6.252	6.886	-0.634
10	7.889	7.854	0.035
12	5.429	5.770	-0.341
14	7.953	7.244	0.709
A1	7.522	8.222	-0.700
A4	7.845	7.745	0.100
A9	8.028	7.745	0.283
A23	8.445	8.097	0.348
A24	8.007	7.959	0.048
A29	8.173	8.097	0.076
A37	8.435	8.046	0.389
A44	8.173	7.959	0.214
A45	8.173	7.658	0.515
A46	8.099	7.796	0.303
A72	8.045	8.398	-0.353
A92	8.315	8.523	-0.208
A103	8.021	8.000	0.021

Supplementary Table 5. Details of the data splitting and predictions for the CoMSIA-SIMCA model. The predicted classes of pK_ds for the training set are the five-group cross-validated values.

Training set

ID	Predicted pK_d	Actual pK_d
A-674563	6	6
AG-013736	6	6
BI-2536	4	4
BIBW-2992	5	4
BMS-345541	4	4
BMS-387032	4	4
BMS-540215	6	6
BIBF-1120	7	7
CEP-701	7	7
CHIR-258	7	7
CI-1033	5	5
CP-690550	4	4
Crizotinib	4	4
Dasatinib	5	5
Erlotinib	5	5
GDC-0879	5	5
GDC-0941	4	4
GSK-1838705A	4	4
GSK-461364A	4	4
GSK-690693	4	4
HKI-272	6	6
INCB18424	4	4
JNJ-28312141	6	6
KW-2449	7	7
Lapatinib	4	4
LY-317615	4	4
LY-333531	4	4
MLN-120B	4	4
MLN-8054	4	4
Pazopanib	6	6
PHA-665752	6	6
PD-173955	6	6
PI-103	4	4
PKC-412	5	5
PLX-4720	5	5
PP-242	6	6
R406	5	5
R547	4	4
SB-203580	5	6
SGX-523	4	4

Test set

ID	Predicted pK_d	Actual pK_d
AT-7519	4	4
AZD-2171	5	6
Flavopiridol	4	4
Gefitinib	5	5
Sunitinib	7	7
Vandetanib	5	5

SKI-606	6	7
Staurosporine	7	7
SU-14813	7	7
TAE-684	6	5
TG-100-115	4	4
TG-101348	4	4
VX-680	5	5
VX-745	4	4

Supplementary Figure 1. Correlation between pIC50 and KierA2 or PEOE_VSA_HYD for the molecules with radius below 7.

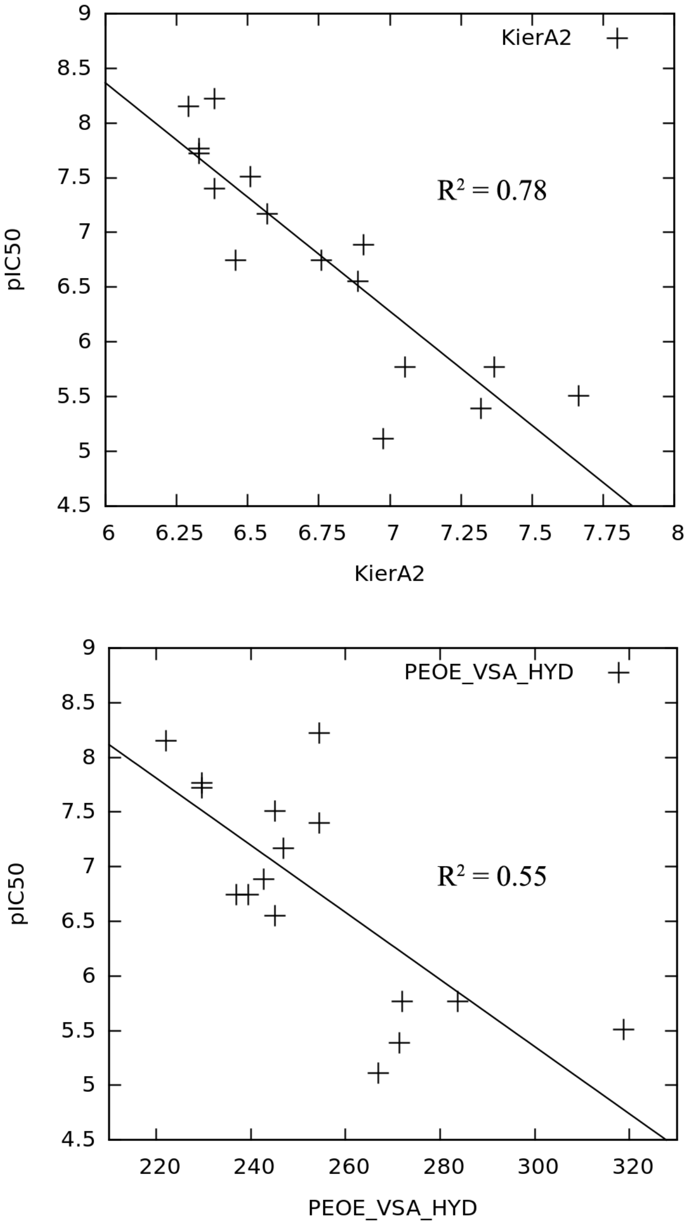

Supplement: Supplementary Information [file srep33534-s1.pdf]
